# Preclinical Insights into the Role of Kir4.1 in Chronic Pain and Depression: Mechanisms and Therapeutic Potential

**DOI:** 10.3390/biom15020165

**Published:** 2025-01-23

**Authors:** Tingfeng Zha, Xinyi Fang, Jiamin Wan, Xiaoyan Chen, Jiu Lin, Qianming Chen

**Affiliations:** Stomatology Hospital, School of Stomatology, Zhejiang University School of Medicine, Zhejiang Provincial Clinical Research Center for Oral Diseases, Key Laboratory of Oral Biomedical Research of Zhejiang Province, Cancer Center of Zhejiang University, Engineering Research Center of Oral Biomaterials and Devices of Zhejiang Province, Hangzhou 310006, China; 22418885@zju.edu.cn (T.Z.); zjussfangxinyi@zju.edu.cn (X.F.); 3210102720@zju.edu.cn (J.W.); ortho_chenxy@zju.edu.cn (X.C.)

**Keywords:** the inwardly rectifying potassium channel 4.1, pain, mental health disorders, glial cells, comorbidity, preclinical studies

## Abstract

Chronic pain and mental health disorders, such as depression and anxiety, frequently co-occur and share underlying mechanisms involving neuronal excitability and synaptic transmission. The inwardly rectifying potassium channel 4.1 (Kir4.1), predominantly expressed in glial cells, is crucial for maintaining extracellular potassium and glutamate homeostasis. Dysregulation of Kir4.1 leads to altered neuronal activity, contributing to both chronic pain and mental health disorders. In chronic pain, downregulation of Kir4.1 impairs potassium buffering and glutamate clearance, increasing neuronal excitability and enhancing pain signaling through peripheral and central sensitization. In mental health disorders, impaired Kir4.1 function disrupts neurotrophic factor secretion and neuroinflammatory pathways, leading to mood disturbances. This review primarily summarizes findings from preclinical studies to examine the relationship between Kir4.1 and the pathogenesis of chronic pain and mental health disorders, discussing its molecular structure, expression patterns, and functional roles. Furthermore, we explore therapeutic strategies targeting Kir4.1, including pharmacological modulators and gene therapy approaches, emphasizing its potential as a novel therapeutic target.

## 1. Introduction

Pain is a multidimensional, unpleasant sensation categorized into acute and chronic forms, based on duration and underlying cause. Chronic pain, defined as pain that persists for more than three months, is recognized as a major public health issue, affecting over 30% of the global population and resulting in substantial personal and socioeconomic impacts [1]. This condition is commonly debilitating and is frequently accompanied by mental health disorders, such as depression and anxiety [2,3,4]. Studies indicated that up to 60% of individuals with chronic pain also received a depression diagnosis, and a comparable proportion met the diagnostic criteria for anxiety [5]. In a multicenter cohort study of 182 chronic pain patients, 20.3% were diagnosed with anxiety disorders and 29.7% with mood disorders [6]. Moreover, the prevalence of major depressive disorder (MDD) ranged from 2% to 61% among populations with chronic pain, and exceeding 50% in patients with fibromyalgia, temporomandibular joint disorders, chronic spinal pain, and chronic abdominal pain [5]. Similarly, the estimated current or 12-month prevalence of high levels of anxiety or anxiety disorders also surpassed 50% [5]. These data suggest a strong association between chronic pain and mental health disorders. Several brain regions, including the anterior cingulate cortex (ACC), medial prefrontal cortex (mPFC), dorsal raphe nucleus (DRN), nucleus accumbens (NAc), central amygdala (CeA), ventral striatum (VS), and periaqueductal gray matter, are associated with the comorbidity of mental health disorders and chronic pain [2,7,8,9], where various glial cells likely play crucial roles.

Glial cells, including astrocytes, microglia, and satellite glial cells (SGCs), serve as key factors in the pathogenesis of chronic pain and mental health disorders through their interactions with neurons and their ion channels [10]. In the central nervous system (CNS), astrocytes and microglia become reactive in response to injuries or pathological conditions, undergoing morphological, molecular, and functional changes that contribute to chronic pain [11]. These reactive glial cells release pro-inflammatory cytokines and other mediators that modulate neuronal activity and pain sensitivity [12,13]. Ion channels, such as voltage-sensitive sodium, calcium, and potassium channels, as well as non-selective gap junctions and hemichannels, are crucial in the process of neuropathic pain becoming chronic by facilitating intercellular communication and altering the extracellular microenvironment [12,14]. Specifically, the downregulation of Kir4.1 in SGCs has been linked to increased neuronal activity and pain in various rodent models, suggesting a potential therapeutic target [15,16]. Moreover, glial cells are involved in the regulation of ionic homeostasis and synaptic plasticity, which are essential for maintaining neuronal function and responding to brain injuries [14]. The dysregulation of these processes can lead to neuroinflammation and excitotoxicity, further exacerbating chronic pain and contributing to mental health disorders [14,17]. Understanding the complex interplay between glial cells, their ion channels, and neuronal networks is crucial for developing new therapeutic strategies to manage chronic pain and associated mental health conditions.

Inwardly rectifying potassium (Kir) channels, characterized by limited outward flow during membrane depolarization and increased inward flow during hyperpolarization, play significant roles in regulating neuronal excitability and synaptic function, which are crucial in chronic pain and mental health disorders [18,19]. Classical Kir2.x channels, expressed in pacemaker neurons within the superficial dorsal horn, contribute to pain transmission by regulating spontaneous pacemaker activity [20]. The downregulation of adenosine triphosphate-sensitive potassium (KATP) channels (Kir.6x) in dorsal root ganglion (DRG) neurons and Schwann cells is linked to neuropathic pain [21], as evidenced in bone cancer pain models where the loss of Kir6.2 subunits worsens pain sensitivity [22], while activating the Kir6.1 on astrocytes alleviates postoperative pain through the JNK/MCP-1 pathway [23]. Furthermore, Kir3.1 channels modulate opioid analgesia by regulating GABAergic neuron activity [24]. Beyond pain modulation, Kir channels are also implicated in mental health disorders, such as depression. Kir6.2 knockout models exhibit increased immobility time of both the forced swim test (FST) and tail suspension test (TST), while activation of KATP channels attenuates these behaviors [25]. Additionally, inhibition of Kir3 channels has been linked to the antidepressant effects of tricyclic drugs and Paroxetine [26], while overexpression of Kir2.1 in the NAc is associated with anxiety and depressive phenotypes [27]. These findings underscore the integral role of Kir channels in the pathophysiology of pain and comorbid mental health conditions.

Recently, Kir4.1 has emerged as a novel target for addressing pain and mental health disorders. Predominantly expressed in glial cells within the CNS, Kir4.1 plays a role in maintaining extracellular potassium homeostasis, stabilizing astrocyte resting membrane potential, and facilitating glutamate uptake [28]. Kir4.1 dysfunction is linked to several neurological and psychiatric conditions. For instance, mutations in *KCNJ10* are associated with seizure susceptibility, autism spectrum disorders, and intellectual disabilities, suggesting that altered potassium buffering by astrocytes contributes to neuronal hyperexcitability and synaptic dysfunction [29,30]. Additionally, Kir4.1 dysfunction is implicated in epilepsy pathophysiology; its inhibition increases brain-derived neurotrophic factor (BDNF) expression in astrocytes, potentially contributing to epileptogenesis and other neuropsychiatric disorders [30]. The therapeutic potential of targeting Kir channels, including Kir4.1, is underscored by their involvement in various neurologic and psychiatric conditions such as mood disorders, schizophrenia, and neurodegenerative diseases [28,31]. Moreover, the development of selective inhibitors for Kir4.1/Kir5.1 channels, like VU6036720, emphasizes ongoing efforts to explore these channels as therapeutic targets [32]. Overall, the critical role of Kir4.1 in maintaining neuronal and glial function underscores its importance in the etiology and potential treatment of chronic pain and mental health disorders. This review commences with an examination of the structure, properties, expression patterns, and functions of Kir4.1. Subsequently, we discuss the correlation between Kir4.1 and pain and mental health disorders, exploring the underlying mechanisms and therapeutic strategies targeting Kir4.1, with the aim of providing new insights into novel therapeutic approaches for pain and associated mental health disorders.

## 2. Molecular Structure and Channel Properties of Kir4.1

**Figure 1:**
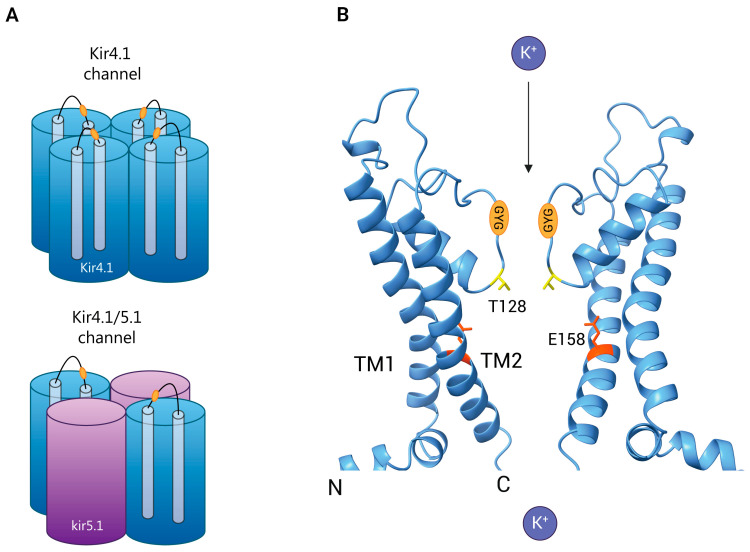
The molecular structure of Kir4.1. (**A**) Kir4.1 is composed of four Kir4.1 subunits, while Kir4.1/5.1 consists of two Kir4.1 subunits and two Kir5.1 subunits. (**B**) The Kir4.1 subunit contains two transmembrane regions, an extracellular pore-forming loop, and intracellular N- and C-terminal domains. The pore-forming region features a G-Y-G motif that is responsible for selective K^+^ permeation. Amino acid residues T128 and E158 serve as binding sites for certain antidepressants and small-molecule compounds that block Kir4.1. E158 is located at the center of the TM2 region, while T128 resides in the inner pore region below the G-Y-G sequence. The schematic was made using BioRender (https://app.biorender.com/, accessed on 18 November 2024).

The Kir family now consists of 15 members, divided into four functional categories: (1) G protein-gated channels (Kir3.x), (2) ATP-sensitive channels (Kir6.x), (3) classical channels (Kir2.x), and (4) K^+^ transport channels (Kir1.x, Kir4.x, Kir5.x, and Kir7.x) [28] (Table 1). Kir4.1, a subtype of K^+^ transport channel, exists in two configurations: a homotetramer composed entirely of Kir4.1 subunits (Kir4.1) and a heterotetramer consisting of two Kir4.1 and two Kir5.1 subunits (Kir4.1/Kir5.1) [33,34] (Figure 1). The *KCNJ10* gene is responsible for encoding the Kir4.1 subunit. In humans, this gene is located on chromosome 1 (1q23.2), in mice on chromosome 1 (1H3), and in rats on chromosome 13 (13q24), encoding proteins of 379, 476, and 381 amino acids, respectively [18,35]. Each subunit has two transmembrane (TM) regions, an extracellular pore-forming domain, and intracellular N-terminal and C-terminal domains; the G-Y-G signature sequence within the pore-forming region is essential for selective K^+^ ion passage [18] (Figure 1). Research has demonstrated that Kir4.1 exhibits inward rectification, allowing large inward K^+^ currents but minimal outward K^+^ currents [18,35]. Several antidepressants, including nortriptyline, sertraline, and fluoxetine, block Kir4.1 by interacting primarily with two amino acid residues: E158 in the TM2 region and T128 adjacent to the G-Y-G motif [36]. E158 is centrally positioned within the TM2 region, while T128 is situated in the inner pore beneath the G-Y-G motif (Figure 1). Moreover, pharmacophore modeling and molecular docking studies indicate that T128 forms hydrogen bonds with the benzene ring of antidepressants, while E158 establishes ionic interactions with the amine group [36]. Further studies estimate the central cavity of Kir4.1 to have a volume of approximately 220 Å^3^ in the closed state and 360 Å^3^ in the open state, sufficient to accommodate antidepressants such as fluoxetine (213 Å^3^) [36]. The elucidation of the Kir4.1 structure establishes a solid foundation for advancing research into its channel dynamics, biochemical functions, and pharmacological interactions.

## 3. The Role of Kir4.1 in the Nervous System

### 3.1. The Expression of Kir4.1 in the Nervous System

**Figure 2:**
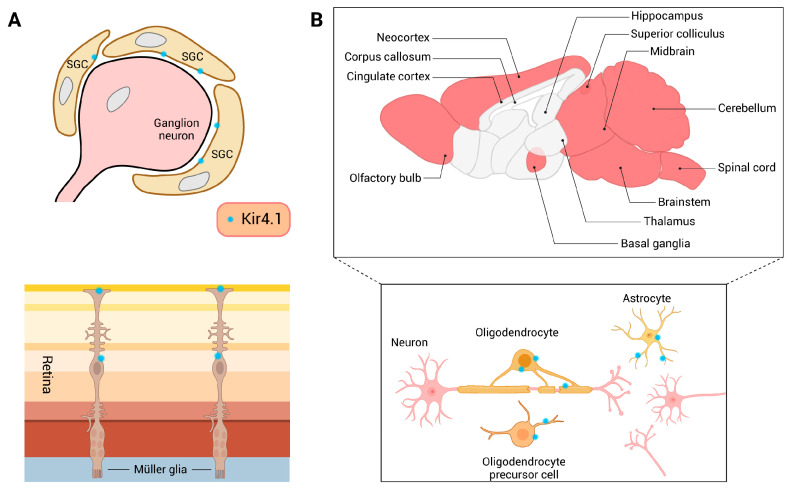
The expression profile of Kir4.1 in the rat nervous system. (**A**) Kir4.1 is mainly expressed in SGCs and Müller glia in the peripheral nervous system. (**B**) As for the CNS, Kir4.1 is mainly distributed in astrocytes, OLs, and OCPs. Moreover, the levels vary significantly across different regions, with notably higher abundance in areas such as the cerebellum, brainstem, spinal cord, hippocampus, and olfactory bulb. Note: In the upper section of (**B**), the red coloration simply indicates that Kir4.1 expression levels are higher in these brain regions compared to others. SGC: satellite glial cell. The schematic was made using BioRender (https://app.biorender.com/, accessed on 18 November 2024). The helical structure representing the Kir4.1 subunit in (**B**) was generated by ChimeraX-1.9 (San Francisco, CA, USA).

Findings in rodents have confirmed the expression of Kir4.1 in various regions of the CNS, including the olfactory bulb, cerebellum, brainstem, hippocampus (HIP), thalamus, neocortex, spinal cord, and midbrain, as well as in the peripheral nervous system, which includes the retina and ganglia [28,37]. However, Kir4.1 expression varies among brain regions, with levels in the basal ganglia and superior colliculus being several to ten times higher than those in the corpus callosum, HIP, and cingulate cortex [28,38] (Figure 2). Notably, Kir4.1 expression is robust in the glomerular layer of the olfactory bulb, Bergmann glia in the cerebellum, and Müller glia, yet it is less prominent in white matter regions like the corpus callosum [37,39]. At the cellular level, Kir4.1 is expressed on astrocytes, radial glia, Bergmann glia, retinal Müller glia, SGCs, oligodendrocytes (OLs), oligodendrocyte precursor cells (OPCs), non-myelinating Schwann cells, etc. [28,35,40]. In particular, the function of Kir4.1 in astrocytes has garnered increasing focus due to its implications in neurological disorders [35]. Although Kir4.1 is expressed in various cell types across the nervous system, its expression shows distinct temporal and spatial patterns intricately associated with age, brain regions, and cell types. Studies indicate that Kir4.1 expression in the brain undergoes significant upregulation during the early postnatal period, with levels on postnatal day 28 being several times higher than those on day 14 [28,41]. Additionally, in pathological conditions like epilepsy, Kir4.1 levels are significantly reduced in specific brain regions, such as the occipitotemporal cortex and thalamus, contributing to neuronal hyperexcitability [42]. Overall, the expression and regulation of Kir4.1 across different regions of the nervous system highlight its vital role in maintaining neural homeostasis and its potential as a therapeutic target for various neurological conditions.

### 3.2. The Functions of Kir4.1 in the Nervous System

**Figure 3:**
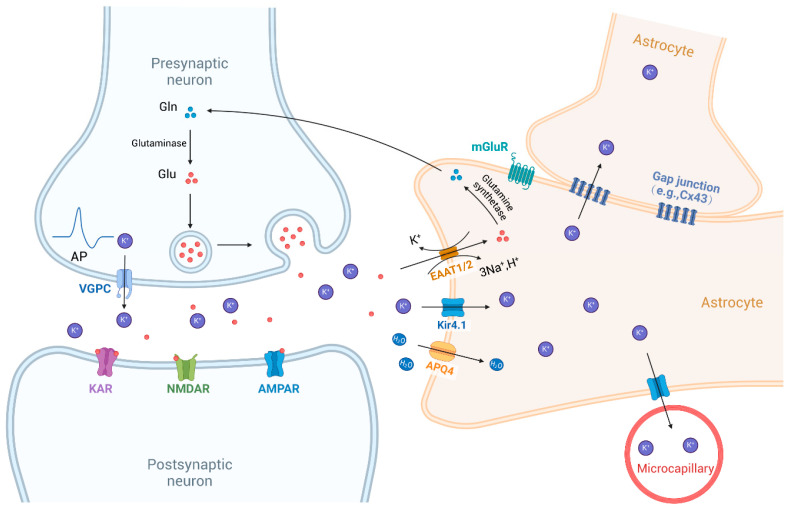
The spatial K^+^ buffering mediated by Kir4.1. During the repolarization phase of action potentials, neurons release a substantial amount of K^+^. Astrocytes absorb the excess K^+^ through Kir4.1 and redistribute it to microcapillaries or transfer it through gap junctions to other astrocytes with lower K^+^ concentrations, a process termed “spatial K^+^ buffering.” Furthermore, Kir4.1 co-localizes with EAATs (e.g., EAAT1 and EAAT2) and AQP4, coupling with astrocytic uptake of glutamate and water. Gln: glutamine, Glu: glutamate, AP: action potential, VGPC: voltage-gated potassium channel, KAR: kainic acid receptor, NMADR: N-methyl-D-aspartate receptor, AMPAR: α-amino-3-hydroxy-5-methyl-4-isoxazolepropionic acid receptor, mGluR: metabotropic glutamate receptor. The schematic was made using BioRender (https://app.biorender.com/, accessed on 18 November 2024).

Firstly, Kir4.1 exhibits strong inward rectification due to selective blockade by intracellular Mg^2+^ and polyamines, which allows it to maintain high resting K^+^ conductance and hyperpolarized membrane potentials [18]. This feature is essential for K^+^ buffering and stabilization of the extracellular K^+^ concentration ([K^+^]_o_), particularly during neuronal excitation [19]. Following neuronal repolarization, elevated extracellular K^+^ is rapidly taken up by astrocytes through Kir4.1 and redistributed to areas with lower K^+^ levels, such as adjacent astrocytes and microcapillaries, thus preserving K^+^ homeostasis [28]. Connexin 43 (Cx43) is a crucial protein for astrocyte coupling, forming gap junctions that facilitate intercellular communication. Impairment of the Cx43 function can weaken these gap junctions and indirectly affect spatial K^+^ buffering [43]. Secondly, Kir4.1’s role in spatial K^+^ buffering is functionally coupled with glutamate homeostasis. By supporting the electrochemical gradients required for excitatory amino acid transporters (EAATs), Kir4.1 facilitates glutamate clearance from the synaptic space [44]. In models with Kir4.1 knockdown, knockout, or pharmacological inhibition, reduced Kir4.1 function led to a reduction in astrocytic glutamate uptake by 57.0%, 50.0%, and 30.5%, respectively [45,46]. Thirdly, Kir4.1 is implicated in BDNF regulation, where its inhibition enhances BDNF secretion via the activation of the Ras/ERK/CREB signaling pathway [47]. Moreover, Kir4.1 modulates glial cell volume, partly through interaction with aquaporin-4 (AQP4), indicating coordinated regulation of K^+^ and water transport [28]. Disruptions in Kir4.1 expression or localization are linked to glial swelling, as seen in retinal Müller cells following ischemic events [48]. Furthermore, Kir4.1 influences glial cell proliferation and maturation, where decreased Kir currents promote cell cycle progression, while increased currents support cellular maturation [49]. Overall, Kir4.1 is integral to the regulation of ion homeostasis, neurotransmitter clearance, cellular volume, and glial cell function, emphasizing its relevance in both neurophysiological and pathological conditions (Figure 3).

#### 3.2.1. Kir4.1 in Astrocytes

Kir4.1 in astrocytes plays a significant role in regulating the secretion of various neurotrophic and certain inflammatory factors, which are crucial in the progression of neurological diseases. For instance, siRNA-mediated downregulation of Kir4.1 expression or its inhibition by antidepressants increased BDNF expression in astrocytes via activating the Ras/Raf/MEK/ERK pathway [30]. This mechanism may contribute to epileptogenesis and pain sensitization, while activation of Kir4.1 may be involved in the pathogenesis of depressive disorders [35]. The research further confirmed that Kir4.1 downregulation slightly, but significantly, elevated the mRNA expression of glial cell line-derived neurotrophic factor (GDNF) and ciliary neurotrophic factor, although their protein levels were not examined [30]. Kir4.1 is closely related to some inflammatory factors. Both IL-1β and IL-6 inhibit the expression of Kir4.1 [50,51]. By contrast, downregulation of Kir4.1 in astrocytes activates the NLRP3 inflammasome through the NMDAR/calpain-1 signaling pathway in lipopolysaccharide (LPS)-treated mouse [52], while the NLRP3 inflammasome facilitates the conversion of proIL-1β into IL-1β [53], which creates a vicious cycle, finally leading to depression-like behaviors.

Moreover, Kir4.1 is implicated in regulating astrocyte volume through its interaction with AQP4, a water channel that co-localizes with Kir4.1 at the astrocytic endfeet [54]. When exposed to 30% hypotonic solution, astroglial somata swelled, while processes or endfeet remained unaffected [54], possibly due to higher Kir4.1 expression in these regions [39]. With Ba^2+^ application, processes also exhibited significant swelling, underscoring Kir4.1’s role in volume regulation. This aligns with findings in Kir4.1 knockout mice, where both somata and processes displayed markedly swelling under hypotonic conditions, and Ba^2+^ had no additional effect [54]. In addition, in receptors for activated C kinase 1 (RACK1) conditional knockout (cKO) mice, due to the lack of translational inhibition of Kir4.1 by RACK1, Kir4.1 levels in astrocytes were significantly elevated. Specifically, RACK1 cKO astrocytes displayed an approximate 2-fold increase in the amplitude of Kir4.1-mediated currents compared with RACK1^fl/fl^ astrocytes. Three-dimensional reconstructions revealed increased volume and elongated processes, potentially driven by enhanced K^+^ currents through Kir4.1 [55].

As the main channel for clearing extracellular K^+^ in astrocytes, Kir4.1 may potentially modulate neuronal firing patterns by influencing [K^+^]_o_ [56]. Previous research identified Kir4.1 as a key regulator of neuronal rhythmicity within the spinal cord central pattern generator (CPG). In in vitro experiments on lumbar spinal cord preparations, Ba^2^^+^ was used to block Kir4.1 currents, which led to approximately 70% of TTX-sensitive neuronal oscillations transitioning from burst firing to tonic firing, and around 85% of the burst activity in TTX-insensitive neuronal oscillations ceased, which meant a slowed locomotor rhythm [57]. Consistent with these findings, Kir4.1 knockdown mice exhibited reduced motor activity levels and impaired motor performance in mice during challenging tasks [57]. Moreover, the loss of Kir4.1 function in the hippocampus led to increased neuronal gamma oscillation power and enhanced network synchrony, which are considered fundamental mechanisms underlying epilepsy [58]. Prior research reported that Kir4.1 upregulation triggers burst firing in lateral habenula (LHb) neurons, which was highly associated with depressive-like behaviors [59]. In contrast, the upregulation of Kir4.1 in hippocampal astrocytes reduced the frequency and duration of neuronal burst firing [55]. Additionally, targeted Kir4.1 knockout or knockdown in spinal astrocytes increased the proportion of neurons exhibiting sustained firing, which may contribute to pain maintenance. For example, under baseline conditions (0-pA injection) and moderate current injection conditions (30-pA and/or 60-pA), the proportion of tonic neuronal firing in the Kir4.1-siRNA-treated group was 23%, 77%, and/or 69%, respectively. In contrast, the corresponding proportions in the control group were 5%, 70%, and/or 50% [60]. In summary, Kir4.1 induces distinct neuronal firing patterns in different regions, suggesting that its role in regulating neuronal activity may be region-specific.

The resting membrane potential is intricately linked to proliferative potential; cells in the division phase typically exhibit a relatively depolarized membrane potential, while cells with a hyperpolarized resting membrane potential rarely enter mitosis [61]. Given that Kir4.1 is essential in establishing resting membrane potential, Kir4.1 also regulates the cell cycle and maturation of astrocytes [28]. Research indicated that the upregulation of Kir channels hyperpolarized the cell membrane, prompting astrocytes to exit the cell cycle [61]. Furthermore, inhibiting Kir4.1 with Ba^2^^+^ markedly increased the proportion of S-phase cells from 5% to 26% and delayed astrocyte maturation [62]. A similar phenomenon occurred in gliomas, where cells exhibited relatively depolarized resting membrane potentials, which were associated with the mislocalization of Kir4.1 [63]. Furthermore, overexpression of Kir4.1 drove a significant shift of cells from the G2/M phase to the quiescent G0/G1 phase, resulting in a 37.2% reduction in cells in the G2/M phase within 5 days [49]. These findings indicate that Kir4.1 may be a promising target for cancer therapy.

Moreover, Kir4.1 in the retrotrapezoid nucleus (RTN) astrocytes may regulate their sensitivity to CO_2_/H^+^ and affect respiratory drive. Previous research demonstrated that inhibition of Kir4.1/5.1 by H^+^ led to astrocyte depolarization, which may facilitate ATP release and subsequently activate RTN chemoreceptors [64]. However, a more recent finding presents conflicting results: astrocyte-specific inducible knockout of the *KCNJ10* gene in mice led to a diminished respiratory response to high CO_2_ levels, whereas the re-expression of Kir4.1 in RTN astrocytes partially restored this function [65]. At the cellular level, these changes were characterized by reduced sensitivity to CO_2_/H^+^ and weakened paracrine signaling to respiratory neurons [65]. The underlying cause of this discrepancy remains unclear; nonetheless, it is evident that Kir4.1 is involved in regulating respiratory drive and may be associated with respiratory dysfunctions in certain neurological disorders, such as keratitis-ichthyosis-deafness syndrome and Rett syndrome.

#### 3.2.2. Kir4.1 in Oligodendrocytes and Related Cells

Oligodendrocytes and oligodendrocyte precursor cells belong to the same cell lineage, so they are discussed together in this section.

Myelin basic protein (MBP) is a key myelin sheath structural protein unique to mature OLs [66], and Kir4.1 is associated with its formation. For instance, shRNA-mediated Kir4.1 knockdown, as well as blockade by desipramine and Ba^2^^+^, decreased the proportion of MBP-positive OLs by 51%, 61%, and 36%, respectively. Further study revealed that inhibiting Kir4.1 did not affect OPC proliferation, cell cycle, or survival [67], which was consistent with previous research findings [68]. However, another study indicated that the absence of Kir4.1 in OPCs led to an early exit from the cell cycle and accelerated myelination [69]. Although some conclusions differ, it is certain that Kir4.1 inhibition impacts OPC differentiation and OL maturation, which is also confirmed by in vivo experiments [70]. The team has also pointed out the mechanism by which Kir4.1 regulates OPC differentiation: Kir4.1 inhibition not only affects OPC differentiation by raising intracellular pH via the sodium/hydrogen exchanger, but also by reducing glycogen synthase kinase 3β activity and downregulating SRY-box transcription factor 10, both of which promote OPC differentiation [67]. However, it has also been reported that Kir4.1 deficiency only mildly affects the proliferation and differentiation of OPCs in the HIP and promotes the expression of MBP [71]. These findings significantly diverge from earlier results, possibly due to variations in experimental conditions and sample selection.

OLs are the key cells responsible for myelination, and increasing evidence indicates that Kir4.1 contributes to OL myelination and the pathogenesis of various demyelinating diseases. For example, a study involving 47 patients with acquired demyelinating disease found serum antibodies against Kir4.1 in 57.45% patients but absent in 62 with other neurologic disease or autoimmune disease or the healthy controls [72]. Furthermore, in multiple sclerosis and experimental autoimmune encephalomyelitis, inflammatory demyelination was accompanied by a sustained downregulation of Kir4.1 channels [73]. Various animal models also validate the link between Kir4.1 and demyelinating pathology. Kir4.1 knockout mice showed marked spinal cord hypomyelination, and OLs exhibited morphological immaturity, characterized by a rounder shape, fewer branches, and diminished interactions with other cells [74]. In addition, in the transient middle cerebral artery occlusion (tMCAO) model, Kir4.1 currents in OPCs on the ischemic side decreased by 87% and 97.86%, respectively, indicating impaired Kir4.1 function following ischemia. Demyelination was also observed through transmission electron microscopy [75,76]. Furthermore, cKO of Kir4.1 in OPCs mimicked the thinning of axonal myelin on the lesioned side in mice, and activation of Kir4.1 with pharmacological agents subsequently induced axonal remyelination [76]. These findings directly demonstrate the importance of Kir4.1 channels in the oligodendrocyte lineage for myelination. However, the specific mechanisms underlying these effects remain unclear, potentially related to Kir4.1’s influence on OPC differentiation and OL maturation. Nonetheless, the evidence suggests that Kir4.1 is a promising therapeutic target for various demyelinating conditions.

Moreover, in mature OLs, Kir4.1 is predominantly expressed near axons and is critical for axonal function and integrity, both in adulthood and after white matter injury. Conditional deletion of Kir4.1 in OLs leads to axonal degeneration and mitochondrial damage [69].

#### 3.2.3. Kir4.1 in SGCs

Satellite glial cells, situated in the peripheral nervous system, closely envelop neuronal cell bodies in structures such as the trigeminal ganglion (TG) and DRG. The distance between SGCs and neuronal cell bodies is only about 20 nm, allowing for close communication between them. As a result, this close association leads to the designation of the neuron and its surrounding SGCs as neuron-glial units [15].

SGCs usually serve as insulators between adjacent neurons, but in sensory ganglia, a phenomenon known as “cross-depolarization” occurs, where electrical activity in one neuron triggers depolarization in a neighboring one. This phenomenon is significantly heightened following nerve injury and inflammation, suggesting its relevance to pain signaling [15]. There is substantial evidence indicating that SGCs play a role in cross-depolarization [15], with Kir4.1 recognized as a crucial mediator in this mechanism. Under pathological conditions, diminished Kir4.1 leads to decreased K^+^ uptake by SGC(a), causing elevated [K^+^]_o_ and subsequent SGC(a) depolarization. This depolarization spreads to adjacent SGC(b) through gap junctions, and then SGC(b) releases K^+^ through Kir4.1, which further depolarizes neurons within the same unit—hence the term cross-depolarization [77].

The role of Kir4.1 in neuropathic pain has been well established. Research showed that exposure of SGCs to cisplatin led to a significant reduction in Kir4.1 protein levels, accompanied by increased ROS, which in turn promoted the release of TNF-α and IL-6 [78]. Although this study did not directly investigate the relationship between Kir4.1 downregulation and increased ROS levels, previous research reported that elevating intracellular potassium concentrations in auditory cells could reduce ROS production and mitigate cisplatin’s side effects [79]. A possible explanation is that cisplatin downregulates Kir4.1, a key ion channel regulating K^+^ levels, leading to higher ROS levels and activation of downstream pathways like ERK1/2 and JAK/STAT3. These cascades stimulate the release of TNF-α and IL-6 from SGCs, contributing to chemotherapy-induced peripheral neuropathy. A recent study confirmed that Kir4.1 downregulation activated the ROS-P38 MAPK pathway, thereby promoting pannexin3 expression and contributing to orofacial neuropathic pain [80].

SGCs, which envelop neuronal cell bodies in sympathetic ganglia, are essential for the metabolism, survival, and function of these neurons. SGCs regulate sympathetic neuron activity via a Kir4.1-dependent mechanism. The knockout of Kir4.1 in SGCs enhanced sympathetic neuron activity and was associated with reduced mTOR signaling, cell atrophy, decreased noradrenergic enzyme expression, and neuronal apoptosis [81]. Notably, in spinal motor neurons, selective depletion of Kir4.1 in astrocytes also caused impairments of mTOR signaling and reduced cell body size, yet no cell death was observed [82].

Moreover, Kir4.1 is highly expressed in SGCs of the spiral ganglion and is responsible for removing excess K^+^ generated during neuronal excitation, which is crucial for normal hearing [83]. On one hand, the expression of Kir4.1 in SGCs coincided with the maturation of auditory function, indicating that Kir4.1 is critical for the development of cochlear auditory function. On the other hand, dysfunction or loss of Kir4.1 expression led to depolarization of SGCs, which subsequently affected neurons in the spiral ganglion, ultimately resulting in hearing loss [83]. A study reported reduced Kir4.1 expression in satellite cells of the spiral ganglion in aged mice and humans, suggesting that cochlear SGC dysfunction contributed to age-related neural hearing loss [84]. In summary, SGCs in the spiral ganglion are closely involved in the generation and maintenance of normal hearing.

## 4. Kir4.1 in Pain and Pain-Related Mental Health Disorders

### 4.1. Correlation and Potential Mechanisms Between Pain and Mental Health Disorders

As mentioned above, the comorbidity of pain with depression or anxiety is highly prevalent. This is because the processing of pain signals is extraordinarily complex, activating circuits associated with various mental states including fear, depression, anxiety, and reduced reward [85]. Several brain regions are now known to be associated with the comorbidity of affective disorders in chronic pain, including the ACC, mPFC, DRN, NAc, CeA, VS, and periaqueductal gray matter [7,8,86]. Multiple neural pathways, such as the DRN–CeA–LHb, thalamus–ACC, locus coeruleus (LC)–ACC, bed nucleus of the stria terminalis (BNST)–LH, and VTA–mPFC, have been implicated in the affective changes during the development of chronic pain [7]. Neuroinflammation also plays a significant role in the comorbidity of pain and mental health disorders. Nerve injury-induced neuroinflammation in the spinal cord underlies the development of mental health disorders, and nerve injury leads to region-specific neuroinflammation in the affective forebrain, which subsequently triggers emotional disturbance [87].

#### 4.1.1. The Comorbidity of Pain and Depression

There is a bidirectional relationship between depression and pain: over 50% of chronic pain patients also experience depression, and individuals with depression are twice as likely to develop neuropathic pain [6]. A large population-based study of 118,533 participants further supports this link, showing that individuals with chronic back pain were six times more likely to suffer from depression than those without pain. Conversely, those initially pain-free who later developed depression are at increased risk for chronic back pain. Additionally, the prevalence of depression escalated with the severity of pain [88]. This pattern is mirrored in animal studies, particularly in Wistar–Kyoto (WKY) rats, a well-established model for endogenous depression. In these rats, mechanical allodynia induced by unilateral temporomandibular joint (TMJ) inflammation was notably intensified, and TMJ inflammation also exacerbated depressive behaviors [89]. WKY rats further demonstrated heightened mechanical allodynia following chronic sciatic nerve constriction [90].

Chronic pain and depression share common biological underpinnings, affecting brain regions like the insular cortex, PFC, ACC, thalamus, HIP, and amygdala [91,92]. Depressive hyperalgesia involves a neural pathway originating glutamatergic neurons in the thalamus’s parafascicular nucleus, connecting to GABAergic neurons in the ACC, and ending with glutamatergic neurons [93]. Furthermore, a positive feedback loop between the ACC and VTA (ACCGlu–VTAGABA–VTADA–ACCGlu) contributes to the persistence of pain and related mental health disorders. Disruption of this loop has been shown to significantly alleviate anxiety and depression-like behaviors in mice [7]. In addition, elevated glucocorticoid levels cause structural and functional alterations in the mPFC, HIP, and amygdala—regions implicated in both pain and depressive disorders [94]. Prolonged exposure to corticosterone sufficiently induces depressive-like behaviors and heightened pain sensitivity in mice [95], while chronic stress increases corticotropin-releasing factor expression in CeA, further exacerbating pain [96]. In individuals with depressive disorders, HPA axis abnormalities, including impaired negative feedback regulation, adrenal hypertrophy, hypercortisolism, and glucocorticoid resistance, are also frequently observed [94].

Chronic pain and depression frequently co-occur in patients with chronic inflammatory conditions, such as rheumatoid arthritis or cancer, suggesting that inflammation may serve as a common pathogenic mechanism [97]. On one hand, chronic pain activates microglia and astrocytes, leading to the release of pro-inflammatory cytokines—such as IL-1β, TNF-α, and IL-6—which disrupt synaptic function, heighten pain sensitivity, and exacerbate mental health symptoms [98]. On the other hand, in patients with depression, increased levels of these cytokines negatively impact neuroplasticity and neurogenesis, thereby potentially amplifying pain signaling [98]. Furthermore, neuroinflammation disrupts connectivity between the amygdala and prefrontal cortex, further exacerbating both pain perception and depressive symptoms [99]. Inflammation in the NAc, a central hub for pain perception and emotional regulation, interferes with dopamine signaling, thereby reducing motivation—a core feature of depression [99]. Additionally, the bidirectional relationship between pain and depression can also be understood through pharmacological interventions. Some antidepressants demonstrate notable analgesic properties, as seen with tricyclic antidepressants (TCAs), which are regarded as a gold standard for neuropathic pain management [100]. The efficacy of TCAs is partially attributed to their inhibition of Kir4.1, indicating that Kir4.1 may be crucial in the shared pathophysiology of pain and depression.

#### 4.1.2. The Comorbidity of Pain and Anxiety

Chronic pain patients often experience anxiety as well. Studies indicated that up to 60% of individuals with chronic neuropathic pain suffered from anxiety [5], while the prevalence in patients with rheumatoid arthritis reached 70% [101]. Similar to depression, the link between pain and anxiety is bidirectional. For instance, individuals with migraines were two to three times more likely to develop an anxiety disorder compared to those without migraines, whereas patients with anxiety disorders had twice the risk of experiencing migraines [102]. To investigate the comorbidity of anxiety and chronic pain, various animal pain models have been developed [103]. In neuropathic pain research, chronic nerve compression models—such as partial sciatic nerve ligation, spinal nerve ligation, and chronic constriction injury of the infraorbital nerve (CCI-ION)—are utilized in approximately 90% of studies [103]. For inflammatory pain, complete Freund’s adjuvant (CFA)-induced paw inflammation is the most widely used model [104].

Alterations in specific neural circuits underlie the comorbidity of pain and anxiety. The LHb plays a critical role in regulating both negative emotions and pain responses [105]. In chronic pain conditions, certain neuronal populations within the LHb exhibit increased excitability, a phenomenon frequently linked to intensified negative emotional states, including anxiety and depression [98]. For instance, in chronic neuropathic pain models, nerve injury significantly elevates the spontaneous firing rates of LHb neurons, which correlated strongly with heightened anxiety-like behaviors [106]. Moreover, the LC plays a dual role in this comorbidity. It inhibits pain transmission through descending pathways to the spinal cord while regulating anxiety and emotional responses via ascending pathways to the prefrontal cortex and limbic system [98]. In chronic pain states, LC activity shifts from a pain-inhibitory role to a facilitative one. Following nerve injury, LC neurons undergo lateralized asymmetric plasticity changes, which may enhance both emotional and pain responses, thereby increasing anxiety risk [107]. Furthermore, dopaminergic and GABAergic neurons in the lateral hypothalamus (LH) are implicated in the regulation of both pain and anxiety behaviors. Optogenetic activation of GABAergic neurons in the LH has been shown to induce increased anxiety-like behaviors and elevate pain sensitivity in mice [108].

Similar to its role in depression, neuroinflammation also plays a significant role in the comorbidity of pain and anxiety. The CNS neuroinflammation, often triggered by nerve injury, increases neuronal excitability in forebrain regions like the HIP, amygdala, and prefrontal cortex, which is associated with increased pain sensitivity and anxiety [109]. Peripheral inflammation may also contribute to anxiety development by affecting CNS function. Research indicated that patients with elevated peripheral inflammation levels were more prone to anxiety and depression symptoms [110]. Peripheral inflammatory factors may cross the blood–brain barrier or directly impact the CNS by interacting with vagus nerve endings [87], activating glial cells and triggering the release of pro-inflammatory cytokines, which subsequently amplify the central inflammatory response [110]. Moreover, inflammatory cytokines may disrupt neurotransmitter release in the brain, intensifying the comorbidity of pain and anxiety. Research indicated that elevated levels of TNF and IL-6 decreased the release of dopamine and 5-HT, while IL-1β and TNF in chronic pain states increased glutamate release and reduced GABA synthesis [87]. Additionally, HPA axis dysfunction, reward deficiency, anti-reward imbalance, and disruptions in various brain nuclei, such as the parabrachial nucleus, medial septum, ACC, amygdala, HIP, and BNST, also contribute to the mechanisms underlying the comorbidity of pain and anxiety [99].

In summary, the occurrence of depression or anxiety in chronic pain patients is highly prevalent, and gaining insight into the relationship between these conditions may aid in the treatment of refractory diseases.

### 4.2. Kir4.1 and Pain

In mammals, peripheral lesions in innervated areas activate nerve endings, generating pain signals transmitted along nerve fibers to ganglia, such as the TG or DRG, where they undergo initial processing and integration—a process termed peripheral sensitization. This primary sensory information is subsequently conveyed by neurons to higher centers, including the spinal cord, specific brainstem nuclei, and the cerebral cortex. Within the CNS, these signals are further integrated and amplified, contributing to the phenomenon known as central sensitization [111,112].

Regarding the role of Kir4.1 in peripheral sensitization, accumulating evidence indicates an intrinsic association between Kir4.1 and pain signaling pathways. In neuropathic pain models, such as those induced by CCI-ION and inferior alveolar nerve transection (IANX), a significant downregulation of Kir4.1 expression in SGCs of the TG was observed [16,113]. Conditional knockdown of the *KCNJ10* gene resulted in the manifestation of abnormal orofacial pain behaviors [80,114], whereas restoration of Kir4.1 alleviated allodynia induced by nerve injury, manifested by a higher head-withdrawal threshold [114]. Further studies implicated that diminished Kir4.1 expression activated the ROS-p38 MAPK signaling pathway, leading to an increase in Panx3 protein levels within SGCs of the TG, contributing to the onset of orofacial neuropathic pain [80]. Moreover, orthodontic pain models showed a marked decrease in Kir4.1 mRNA expression in the TG [115]. Intra-TG injection of siRNA targeting Kir4.1 further supported its role, as silencing Kir4.1 induced pain-like behaviors in orofacial regions [16]. Similarly, Kir4.1 function and activity were diminished in SGCs in a paclitaxel-induced neuropathic pain model [116]. Collectively, these findings underscore the critical role of Kir4.1 in the peripheral sensitization mechanisms underlying pain.

**Figure 4:**
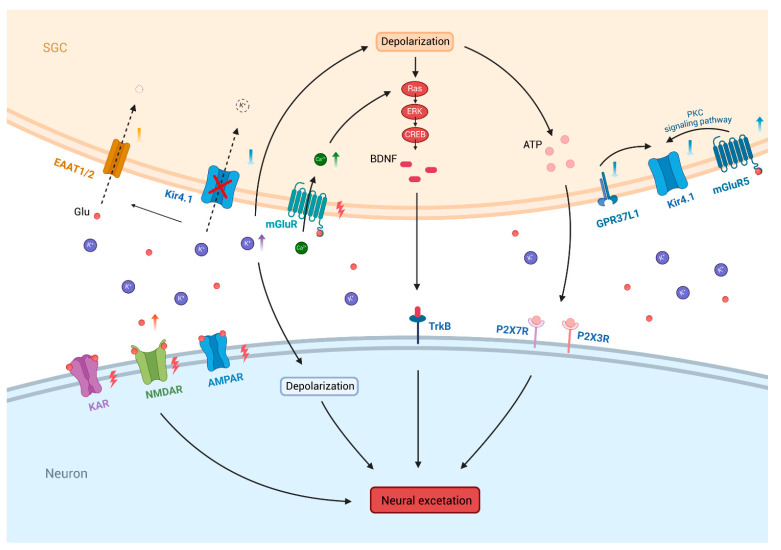
The mechanism by which Kir4.1 on SGCs contributes to pain signal transmission. Following peripheral nerve injury or the application of Kir4.1 inhibitors, the function of Kir4.1 channels decreases, leading to an accumulation of extracellular K^+^. On one hand, the increase in [K^+^]_o_ depolarizes neurons, enhancing their excitability; on the other hand, it causes SGC depolarization, promoting ATP release and activation of the Ras/ERK/CREB signaling pathway, which increases BDNF secretion. These signaling factors then act on P2X7R, P2X3R, and TrkB receptors, further boosting neuronal excitability. Additionally, elevated extracellular glutamate levels increase neuronal excitability directly by activating ionotropic glutamate receptors on neurons and indirectly by binding to mGluR on SGCs. This mGluR activation enhances intracellular Ca^2^^+^ signaling, further stimulating the Ras/ERK/CREB pathway. Together, these processes synergistically promote the transmission of pain signals. The schematic was made using BioRender (https://app.biorender.com/, accessed on 19 November 2024).

However, there is limited research on how pain induces a reduction in Kir4.1. Recent studies showed that after nerve injury, activation of mGluR5 inhibited Kir4.1 via the PKC signaling pathway [114]. G protein-coupled receptor 37-like 1 (GPR37L1) in SGCs was thought to have a protective role in chronic pain, primarily through its regulatory influence on the expression and function of Kir4.1 [116]. The pro-resolving lipid mediator maresin 1 (MaR1), a ligand for GPR37L1, activated the receptor, enhancing potassium ion influx into SGCs via Kir4.1. This mechanism reduced [K^+^]_o_, leading to decreased nociceptor excitability and pain sensitivity [116]. However, in peripheral neuropathy induced by streptozotoxin and paclitaxel, the reduced expression of GPR37L1 led to impaired Kir4.1 function, thereby decreasing the pain threshold [116]. Moreover, GPR37L1 is also expressed in astrocytes [117], and administration of MaR1 into the spinal cord has demonstrated similar analgesic effects, indicating that GPR37L1 mediates a protective role within the CNS as well [116,117] (Figure 4).

Extensive research indicates that Kir4.1 located on SGCs may represent a key regulatory element in modulating neuronal excitability. Kir4.1 plays a critical role in maintaining K^+^ homeostasis, where elevated [K^+^]_o_ results in neuronal depolarization and increased excitability [15]. In addition, the rise in [K^+^]_o_ also depolarizes SGCs, promoting the release of excitatory neurotransmitters like ATP, which activate purinergic receptors such as P2X7R and P2X3R on nearby neurons, further enhancing neuronal excitability and potentially promoting the transmission of pain signals [15,118] (Figure 4). In Kir4.1 transgenic knockout mice, inward K^+^ currents in SGCs are reduced by approximately 50% in Kir4.1^+/−^ mice and become nearly undetectable in Kir4.1^−/−^ mice [119]. These findings suggest that Kir4.1 in SGCs influences pain transmission through the modulation of [K^+^]_o_. Further evidence indicates the glutamate–glutamine cycle is involved in pain mechanisms [120]. Glutamate transporters in SGCs are responsible for adsorbing excess extracellular glutamate, thus preventing accumulation and avoiding excitotoxicity [121]. In astrocytes, Kir4.1 is closely associated with the function of EAATs, regulating extracellular glutamate levels by modulating the potassium gradient and limiting lateral charge transfer (as discussed below). Given the developmental and functional parallels between astrocytes and SGCs [122], it is plausible to hypothesize that in pain models triggered by nerve damage or inflammation, the downregulation of Kir4.1 on SGCs may inhibit EAAT activity, resulting in glutamate accumulation, neuronal excitotoxicity, and consequently contributing to pain. Furthermore, considering the established significance of neurotrophic factors, such as BDNF [123], in the development of pain, and its upregulation in neuropathic pain [113,124]. This suggests that the reduced function of Kir4.1 may elevate [K^+^]_o_, leading to cell membrane depolarization [125], while the increased glutamate levels enhance intracellular Ca^2+^ signaling via activation of metabotropic glutamate receptors [126]. Together, these events could activate the Ras/ERK/CREB pathway, promoting BDNF secretion, thereby enhancing neuronal excitability (Figure 4). Further investigation is required to determine whether Kir4.1-mediated regulation of BDNF in peripheral ganglia plays a role in pain processes and to elucidate the mechanisms involved.

The involvement of Kir4.1 in central sensitization during pain has attracted considerable research interest, particularly concerning its role in astrocytes [28,35]. While the expression and functional significance of Kir4.1 in astrocytes have been studied in various neurological conditions, data specific to pain-related models remain relatively scarce. Current studies indicate that Kir4.1 in astrocytes regulates the secretion of neurotrophic factors, such as BDNF and GDNF, which subsequently influence neuronal excitability and contribute to the progression of pain [35,47]. Recent findings from single-cell RNA sequencing studies indicated that the expression of both Kir4.1 and methyl-CpG-binding protein 2 (MeCP2) was reduced in spinal cord astrocytes following chronic sciatic nerve compression in mice [60]. Moreover, MeCP2 regulated the expression of Kir4.1 in the spinal cord. Electrophysiological studies indicated that Kir4.1 knockdown significantly altered astrocyte excitability and the firing patterns of dorsal spinal cord neurons, with an increase in the proportion of neurons exhibiting sustained firing, which suggested a role for Kir4.1 in the persistence of pain [60]. In summary, preliminary evidence points to the involvement of Kir4.1 in both peripheral and central sensitization associated with pain, and further research is needed to clarify its specific functions and mechanisms across various types of pain conditions.

### 4.3. Kir4.1 and Depression

Depression, affecting approximately 5–20% of the global population, is a widespread mental health disorder characterized by anhedonia, depressed mood, emotional withdrawal, reduced activity, cognitive impairment, sleep disturbances, and psychosomatic symptoms. In severe cases, it is often linked to suicidal behavior [35]. A systematic review suggests that upregulation of Kir4.1 may contribute to the pathogenesis of depression [127]. However, the literature presents conflicting findings: some studies report decreased or unchanged Kir4.1 expression in individuals with depression or in animal models. For example, a postmortem study of 13 patients with depression and 10 healthy controls identified downregulation of *KCNJ10* in the hippocampus, although it remained unclear whether this reduction was a causative factor or a consequence of the depressive state [128]. Furthermore, another study found no significant differences in Kir4.1 protein levels in the PFC, NAc, and HIP compared to control groups [129]. Despite these discrepancies, the prevailing hypothesis links depression pathogenesis to increased Kir4.1 activity or expression. This is supported by findings such as elevated Kir4.1 levels in the postmortem parietal cortex of individuals with MDD [130]. Additionally, treatment with Ginsenoside Rg1 in LPS-induced depressive mice alleviated depressive-like behaviors and facilitated fear extinction, which was associated with Kir4.1 downregulation [131]. Additionally, increased expression of Kir4.1 in astrocytes within the LHb has been observed in two rat models of depression, coinciding with the emergence of depressive-like symptoms [59], which further reinforces the connection between Kir4.1 and depressive pathology.

Kir4.1 may influence the pathogenesis of depression through multiple mechanisms, such as modulating [K^+^]_o_ and glutamate concentration, as well as regulating BDNF secretion [132]. The gain of function of Kir4.1 enhances the uptake of extracellular K^+^, leading to decreased [K^+^]_o_ and glutamate levels. This reduction lowers neuronal excitability and suppresses BDNF secretion, potentially promoting the development of depression [127,132]. Thus, Kir4.1 holds potential as a therapeutic target for the treatment of clinical depression.

**Figure 5:**
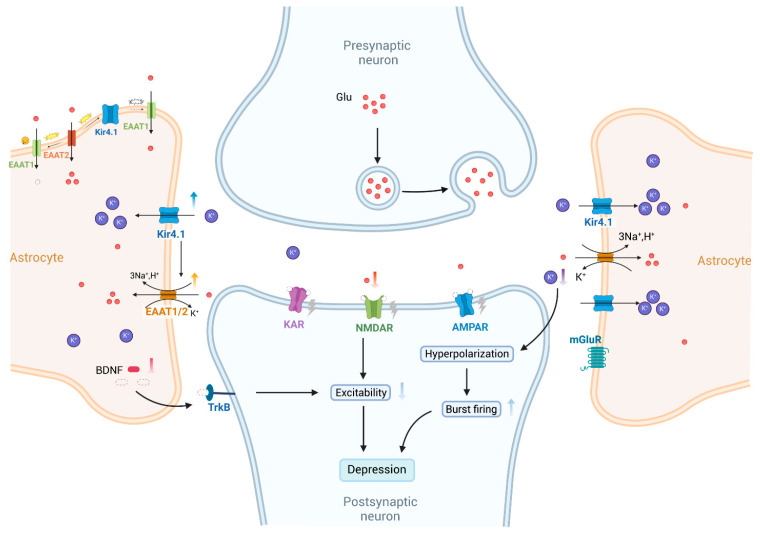
The role of Kir4.1 in the pathogenesis of depression. In depression, Kir4.1 expression is upregulated. On one hand, the increased activity of Kir4.1 leads to a decrease in [K^+^]_o_ and glutamate levels, as well as inhibition of BDNF secretion, ultimately reducing neuronal excitability, which contributes to the development of depression. On the other hand, upregulation of Kir4.1 in LHb induces neuronal hyperpolarization, which in turn triggers burst firing, a process highly associated with depressive-like behaviors. Furthermore, the high conductance property of Kir4.1 can limit lateral charge diffusion generated during glutamate transport. Elevated Kir4.1 expression disrupts this balance, intensifying glutamate uptake by astrocytes and further lowering extracellular glutamate concentration. The schematic was made using BioRender (https://app.biorender.com/, accessed on 19 November 2024).

Ionic homeostasis within the tripartite synapse is fundamental to normal brain function. Specifically, astrocytic Kir4.1-mediated K^+^ spatial buffering is crucial for sustaining the resting membrane potential [35]. Previous research demonstrated that upregulation of Kir4.1 in the LHb induced neuronal hyperpolarization, subsequently triggering burst firing [59], while increased burst activity in LHb neurons was highly correlated with depressive-like behavior in rats [105] (Figure 5). Further studies indicated that ketamine could inhibit Kir4.1-induced burst firing in LHb neurons, leading to the rapid alleviation of depressive symptoms [59,105]. In addition, ketamine elevates extracellular glutamate levels in the PFC and HIP, while enhancing synaptic connectivity via activation of the BDNF-TrkB pathway, thereby mitigating the degenerative changes linked to depression [133,134]. Ketamine also reduces the mobility of Kir4.1 vesicles by regulating intracellular cAMP levels, decreasing the surface density of Kir4.1, which weakens the K^+^ spatial buffering capacity mediated by Kir4.1 [135]. In summary, ketamine exerts its rapid antidepressant effects through multiple mechanisms that likely interact in a non-exclusive manner.

Glutamate, the brain’s principal excitatory neurotransmitter, is crucial for regulating synaptic plasticity and a range of physiological functions, including emotional regulation, cognition, learning, and reward processing [136]. Current evidence indicates that disruption in glutamate metabolism is linked to depression and suicidal behaviors [137]. Astrocytic EAATs are key in maintaining glutamate homeostasis at the tripartite synapse. Astrocytes mainly express EAAT1 and EAAT2, with EAAT2 responsible for approximately 90% of glutamate clearance [35]. In astrocytes, Kir4.1 co-localizes with EAATs to facilitate efficient glutamate transport—a process that is energetically demanding, involving the co-transport of 3 Na^+^, 1 H^+^, and 1 glutamate molecule into the cell, while simultaneously exporting 1 K^+^. Thus, glutamate transport relies not only on transporter expression but also on maintaining an intact electrochemical gradient for the involved ions [28]. The relationship between Kir4.1, glutamate, and depression may be characterized as follows: the gain of function in Kir4.1, resulting in lower extracellular potassium, which in turn reduces neuronal excitability by (1) hyperpolarizing the resting membrane potential of glial cells and (2) enhancing glutamate transport by increasing the driving force (Figure 5). Conversely, this mechanism may also be inferred by examining its reverse effects. When Kir4.1 function was inhibited—using siRNA-mediated knockdown, cKO, or pharmacological inhibitors—the efficiency of astrocytic glutamate uptake was reduced by 57%, 50%, and 30.5%, respectively [45,46]. Moreover, a significant reduction in EAAT1 and EAAT2 expression was observed in the DRG of BTBR mice treated with Kir4.1 inhibitors, such as desipramine and VU0134992 [121], although the precise mechanisms remained unclear. Furthermore, Kir4.1 also helps limit lateral charge transfer within astrocytic processes by providing a high-conductance pathway. This property ensures that electrical signals generated during glutamate transport do not propagate extensively along astrocytic membranes, thus reducing interference with other ion channels and transport proteins, thereby stabilizing glutamate uptake dynamics [138] (Figure 5). However, overexpression of Kir4.1 may disrupt this balance, leading to excessive glutamate uptake by other electrogenic transporters. Curiously, elevated glutamate levels were found in the plasma, cerebrospinal fluid, mPFC, and brain of depression patients [132,139]. This phenomenon might represent a compensatory response aimed at mitigating excitotoxic damage. Despite these insights, further research is necessary to fully elucidate the intricate relationships among Kir4.1, glutamate regulation, and depression pathophysiology.

Brain-derived neurotrophic factor, encoded by the BDNF gene, is essential for regulating synaptic formation and plasticity [140]. BDNF plays a significant role in the pathophysiology of depression and is considered a potential biomarker for its diagnosis [141]. In a corticosterone-induced depressive mouse model, the expression of BDNF was markedly reduced within the dentate gyrus of the HIP [142], and exogenous administration of BDNF markedly alleviated depressive-like behaviors [143]. The upregulation or activation of Kir4.1 reduces BDNF expression, thereby decreasing neuronal excitability, a process associated with the pathophysiology of depression [47] (Figure 5). Furthermore, several antidepressant drugs block Kir4.1 in a subunit-specific manner, promoting BDNF expression through activating the Ras/Raf/MEK/ERK pathway [30]. Interestingly, the relative efficacy of certain antidepressants in inhibiting Kir4.1 correlates with their ability to enhance BDNF secretion (sertraline > fluoxetine > imipramine >> fluvoxamine > mianserin) [35]. This correlation further validates the link between Kir4.1 function and BDNF secretion. Despite these findings, the specific mechanisms through which Kir4.1 modulates the onset and progression of depression via BDNF regulation have not yet been fully elucidated, warranting further investigation.

Neuroinflammation is a significant pathogenic factor in depression, characterized by the excessive secretion of inflammatory cytokines like IL-1β in the brain [144]. The NLRP3 inflammasome has been implicated in the pathophysiology of depression, with its activation associated with the worsening of depressive-like behaviors. Specifically, the mice demonstrate less preference for sucrose than the control group, and longer immobility times in the TST and FST [145]. Research has indicated that in the LPS-induced depressive mouse model, Kir4.1 regulates NLRP3 inflammasome activation via the NMDAR/calpain-1 signaling axis, thereby influencing depressive-like behavior [52]. However, this study overlooked the key role of BDNF, suggesting that specific downregulation of Kir4.1 may alleviate related depressive behaviors by increasing extracellular BDNF levels.

### 4.4. Kir4.1 and Anxiety

Anxiety is an anticipatory emotional state involving the preparation for potential future adverse events. It manifests through symptoms such as worry (cognitive and subjective), avoidance behaviors (observable motor responses), and muscle tension (somatic and autonomic activity) [146]. Preliminary evidence revealed a role for Kir4.1 in anxiety pathogenesis. For example, the dopaminergic (DAergic) system, which is integral to the regulation of emotions, cognition, and social reward processing, has been implicated in anxiety. A recent study found that post-weaning social isolation (PWSI) in male rats increased the excitability of Drd2+ neurons (Drd2+: a type of DA receptor) in the dorsal bed nucleus of the stria terminalis (dBNST), resulting in an altered excitatory-inhibitory balance. Chemogenetic activation of these Drd2+ neurons was sufficient to induce anxiety-like behaviors, whereas overexpression of Kir4.1 hyperpolarized the Drd2+ neurons and significantly reduced anxiety-like behaviors in the PWSI rats [147]. Additionally, in Kir4.1 knockdown mice treated with LPS, compared to the LPS-only group, the number of entries into the lightbox during the light/dark box test was restored, although no significant improvements were observed in behavior during the open field test, elevated plus maze, or 30-min locomotion activity, indicating that conditional knockdown of Kir4.1 has a mild anxiolytic effect [148]. The novelty-suppressed feeding (NSF) test is commonly used to assess anxiety and depressive symptoms by measuring the delay in feeding in a novel environment. Overexpression of Kir4.1 in the LHb leads to a prolonged delay in feeding during the NSF test, while applying Lys05, a Kir4.1 inhibitor, could reverse this effect [149]. These findings suggest that both upregulation and inhibition of Kir4.1 alleviate anxiety-like behaviors. A possible explanation is that depression and anxiety frequently coexist, and inhibiting Kir4.1 might alleviate anxiety while also mitigating depressive symptoms. However, the specific mechanisms by which Kir4.1 regulates anxiety remain unclear, and further research is needed.

### 4.5. Kir4.1 and Aversion

Aversion is characterized by behavioral and emotional responses such as dislike, disgust, and fear, leading to avoidance behaviors and decreasing the probability of engaging in the associated activity [150]. Existing research indicates that the LHb is involved in encoding negative emotions, including aversion and depression [151,152]. Further studies revealed that increased burst firing in LHb neurons was sufficient to drive aversion and depressive states [105]. Kir4.1 played a pivotal role in regulating the burst activity of LHb neurons, and ketamine helped to alleviate aversive emotions by inhibiting this channel [59]. Additionally, targeted silencing of Kir4.1 using dsRNA not only induced facial pain-like behavior but also heightened aversive responses to mechanical stimuli [119]. More recent research indicated that knocking down Kir4.1 in the paraventricular nucleus of the hypothalamus reduced depressive-like behaviors while enhancing the consolidation of aversive memories [148]. Therefore, Kir4.1 appears to be involved in the regulation of aversive emotions. However, the specific mechanisms underlying this relationship are still not well understood, warranting further investigation.

## 5. Treatment Strategies for Pain and Mental Health Disorders or Their Comorbidity Targeting Kir4.1

Kir4.1, predominantly expressed in glial cells, is essential for maintaining potassium and glutamate homeostasis within the tripartite synapse. It also plays a key role in regulating BDNF secretion, as well as in modulating neuronal excitability and synaptic plasticity [28]. Dysregulation of Kir4.1 has been linked to the onset and progression of various neurological and psychiatric disorders, including pain, depression, Alzheimer’s disease, and epilepsy [28]. Preclinical studies have observed the downregulation of Kir4.1 in multiple pain models, including neuropathic pain models established by nerve injury (IANX [113], CCI-ION [16,80]), and inflammatory pain models induced by CFA [121]. Using adeno-associated virus (AAV) to upregulate Kir4.1 expression or employing MaR1 to activate GPR37L1, which indirectly enhanced Kir4.1 function, significantly alleviated or even reverse pain behaviors [114,116]. In addition, the ectopic mechanical allodynia observed in Kir4.1-cKO animals [80,114] further corroborated the critical regulatory role in neuropathic pain. The congenital learned helplessness model [59], the corticosterone-induced depression (CORT) model, and the olfactory bulbectomy (OBX) model [149] are commonly used to simulate the core pathological features of human depression. Studies showed that Kir4.1 upregulation in the LHb triggered neuronal bursting that was positively correlated with depression-like behaviors [59]. By contrast, pharmacological inhibition of Kir4.1 using Lys05 or downregulation of its expression blocked abnormal bursts and alleviated depressive phenotypes, which was evidenced by reduced immobility in the FST and/or TST, increased bar pressing in the learned helplessness test, and increased sucrose consumption in the sucrose preference test [59,149]. Consequently, targeting Kir4.1 offers a promising therapeutic strategy for managing these conditions.

### 5.1. The Drugs Targeting Kir4.1

Several neuropharmacological agents have been found to interact with Kir4.1. Tricyclic antidepressants (e.g., nortriptyline, desipramine, and imipramine) inhibit Kir4.1 currents in a voltage-dependent manner [153] and selective serotonin reuptake inhibitors (SSRIs) in a voltage-independent manner [154]. The relative potency of these antidepressants in inhibiting Kir4.1 is ranked as follows: sertraline > fluoxetine > imipramine > fluvoxamine > mianserin [35]. Electrophysiological and computational analyses of the three-dimensional structures reveal that the inhibitory effect is due to the formation of hydrogen bonds and ionic interactions with channel residues [36]. This suggests that the inhibition of Kir4.1 might contribute to the antidepressant action of TCAs and SSRIs. However, these agents inhibit Kir4.1 at concentrations in the tens of micromolar range [155], which may compromise their specificity and raise the likelihood of off-target effects. Recently, high-throughput techniques have identified more potent small-molecule inhibitors, such as VU0134992 and Lys05, with half-maximal inhibitory concentration (IC50) values of 0.97 µM and 0.22 µM, respectively [149,156]. Due to its high potency and specificity, VU0134992 has been extensively used as a research tool to block Kir4.1, comparable in efficacy to Ba^2+^ [156]. Not only against Kir4.1-driven depressive-like phenotypes but also multiple depression animal models including the NSF test, a CORT model, and an OBX model, a single dose of Lys05 demonstrated antidepressant effects 1 h after administration, with efficacy comparable to that of S-ketamine [149]. Additionally, Kir4.1 is also inhibited by other pharmacological agents, including antimalarial drugs (e.g., quinacrine and chloroquine), pentamidine, and aminoglycoside antibiotics (e.g., gentamicin and neomycin) [35]. Among these, pentamidine—used in treating Pneumocystis carinii pneumonia—emerges as the most effective Kir4.1 blocker, with an IC50 value of 0.097 µM [157]. Notably, similar to antidepressants, these drugs primarily bind to the E158 and T128 residues and their inhibitory effects via interactions with the G-Y-G motif [35]. Moreover, ketamine reduces the cytoplasmic mobility of Kir4.1 vesicles, thereby decreasing their surface density [135]. Inhibition of Kir4.1 results in elevated levels of potassium and glutamate within the tripartite synapse, alongside increased secretion of BDNF [28,35]. These changes enhance neuronal excitability and ameliorate depression-like behaviors, supporting the potential of Kir4.1 blockers as therapeutic agents for depressive disorders (Table 2).

In contrast, for chronic pain, reduction in neuronal excitability is crucial, positioning the enhancement of Kir4.1 activity as a promising therapeutic approach. The glucocorticoid dexamethasone [158] and certain antiepileptic drugs [159], including sodium valproate, phenytoin, and phenobarbital, have been shown to increase Kir4.1 channel expression. Furthermore, Kir4.1 is regulated epigenetically, with agents like azacitidine enhancing its expression [38]. In the tMCAO mouse model, luteolin increases Kir4.1 currents and promotes remyelination, indicating it is a potent Kir4.1 activator of OPCs [76] (Table 2). In summary, although there are no drugs specifically targeting Kir4.1 at present, insights into the modulation of this channel by existing pharmacological agents highlight potential therapeutic strategies for managing pain and related conditions.

**Table 2:** The drugs targeting Kir4.1.

Drugs	Effects on Kir4.1	IC50 Value (Patch Configuration)	Binding Sites	Voltage Dependency	Reference
**TCAs**					
Nortriptyline	Block	38 μM	E158, T128	Yes	[153]
Desipramine		(whole-cell)			
Imipramine					
**SSRIs**					
Sertraline	Block	7.2 μM	E158, T128	No	[154]
Fluoxetine		15.2 μM			
		(whole-cell)			
VU0134992	Block	0.97 μM	E158, I159	Yes	[156]
		(whole-cell)			
Lys05	Block	0.22 µM	T128, G158,	No	[149]
		(whole-cell)	I159		
**A** **ntimalarial drugs**					
Quinacrine	Block	1.8 μM	E158, T128	Yes	[160]
		(inside-out)			
Chloroquine		ca. 0.5 μM			[161]
		(inside-out)			
		ca. 7 μM			
		(whole-cell)			
Pentamidine	Block	0.097 μM	E158, T127,	Yes	[157]
		(inside-out)	T128		
**Aminoglycosides**					
Gentamycin	Block	6.2 μM	E158, T128	Yes	[162]
Neomycin		63.8 μM			
		(inside-out)			
Ketamine	Downregulate	/	/	/	[135]
Dexamethasone	Upregulate	/	/	/	[158]
**A** **ntiepileptic drugs**					
Phenytoin	Upregulate	/	/	/	[159]
Sodium valproate					
Phenobarbital					
Azacitidine	Upregulate	/	/	/	[38]
Luteolin	Upregulate	/	/	/	[76]

### 5.2. Treatment Strategies for Comorbid Chronic Pain and Mental Disorders Targeting Kir4.1

A systematic review and meta-analysis strongly recommend TCAs and serotonin-norepinephrine reuptake inhibitors, pregabalin, and gabapentin as first-line treatments for neuropathic pain [163]. Pregabalin and gabapentin, collectively gabapentinoids, are primarily anticonvulsant drugs. Over the past two decades, they have been increasingly prescribed for pain [164]. Their analgesic effects are believed to stem from binding to the α_2_-δ subunit of voltage-gated calcium channels, which decreases the release of glutamate, norepinephrine, and substance P [165]. SSRIs also exhibit an analgesic effect, although TCAs are generally more powerful [166]. As previously noted, both TCAs and SSRIs interact with Kir4.1 [35], which may contribute to their antidepressant effects. However, the role of Kir4.1 in mediating their analgesic effects remains unclear. Despite their therapeutic benefits, TCAs and SSRIs are associated with cardiovascular side effects, including cardiotoxicity, orthostatic hypotension, and elevated blood pressure [167]. Moreover, these medications have other limitations, such as a delayed onset in relieving mood symptoms and poor efficacy in treatment-resistant depression [168]. Conversely, ketamine is distinguished by its rapid, potent, and long-lasting antidepressant effects [169]. It inhibits Kir4.1-induced bursting activity in the LHb, a process implicated in the pathophysiology of depression [59,105], and reduces the surface density of Kir4.1 [135], suggesting a potential link between these mechanisms. Additionally, ketamine shows promise in treating neuropathic pain and complex regional pain syndrome, although its use carries a high risk of misuse [169,170]. Given their dual therapeutic benefits, TCAs, SSRIs, and ketamine are often employed to manage coexisting chronic pain, anxiety, and depression [166]. Other clinical conditions are also characterized by comorbid pain and mental health challenges. For instance, patients with spinal cord injury frequently experience chronic pain, depression, anxiety, and even suicidal ideation [171,172]. Chronic stress or elevated corticosterone levels downregulate Kir4.1 expression on SGCs in the DRG of spinal cord injury models, which results in the accumulation of ROS and mitochondrial damage, hindering axonal regeneration, while overexpression of Kir4.1 mitigates these effects [173]. Similarly, most multiple sclerosis patients suffer from both pain and depression [174], and autoantibodies targeting Kir4.1 are thought to be significant pathogenic factors [175]. These findings underscore the potential of developing therapeutic strategies targeting Kir4.1 to address both pain and comorbid mental health conditions.

Currently, improving drug specificity and optimizing pharmacokinetics remain major challenges in developing effective therapeutics. TCAs and SSRIs represent two primary classes of antidepressants with distinct mechanisms. TCAs modulate both serotonin and norepinephrine pathways, whereas SSRIs predominantly inhibit serotonin reuptake [166]. These actions form the core of their antidepressant mechanisms, with interactions involving Kir4.1 contributing only partially to their overall effect [153,154]. Despite advances, specificity remains an issue for several small molecules. For instance, VU0134992, a selective inhibitor for homomeric Kir4.1, demonstrates greater potency and specificity compared to other Kir4.1 inhibitors, such as amitriptyline, nortriptyline, and fluoxetine. However, VU0134992 also exhibits activity against other channels, including Kir4.1/5.1, Kir1.1, Kir2.1, and Kir2.2 [156]. ML133, another inhibitor, predominantly targets Kir2.x channels with a potency considerably higher than that for Kir4.1 [155]. The pharmacokinetics of these compounds are also suboptimal. VU0134992 has limited CNS penetration and a short half-life of 10.6 min [156], while ML133 has a high plasma protein binding rate and substantial intrinsic clearance [155]. Recently, high-throughput screening identified Lys05, a new compound showing improved inhibition of Kir4.1 (IC50 = 0.22 µM) with CNS penetration, indicated by a brain-to-plasma ratio of 0.46 and a plasma half-life of 16.7 h [149]. However, Lys05 also lacks complete specificity, affecting other potassium channels such as TWIK1, THIK1, and TREK1 in astrocytes, as well as neuronal KCNQ2, KCNQ3, and cardiac hERG channels [149]. The lack of specificity in Kir4.1 modulators may be attributed to several factors. First, Kir4.1, as a member of the Kir channel family, exhibits a highly conserved structural framework. For instance, the critical binding site T128 on Kir4.1 is similarly conserved across other Kir subunits [176]. Additionally, Kir4.1 forms heterotetramers with Kir5.1 and the physiological properties of Kir4.1/Kir5.1 differ significantly from those of Kir4.1. The presence of this heteromeric form further complicates the specificity of regulation [18].

Molecular docking and site-directed mutagenesis studies have shown that VU0134992 interacts with E158 and I159 of Kir4.1 [156]. Cryo-electron microscopy analysis has further elucidated the interaction, revealing that Thr128, Glu158, and Ile159 are critical molecular determinants for Lys05 binding [149]. Despite the conservation of these residues in Kir4.2, Lys05 does not bind to it, implying that subtle differences in the binding pocket or molecular environment impact binding affinity [149]. In addition, inhibitors interact with Kir channels through diverse mechanisms, including allosteric modulation [177], regulation via phosphatidylinositol 4,5-bisphosphate [178], and direct central pore blockage [149,153,154,156]. Further exploration of these diverse mechanisms could enhance the therapeutic potential of Kir4.1 modulation. Notably, since Kir4.1 co-regulates neuronal activity in LHb alongside NMDA receptors [59,105], targeting Kir4.1 may have synergistic effects when combined with ketamine or similar antidepressants.

However, treatment strategies targeting Kir4.1 are still facing significant challenges. Firstly, systematic administration of specific Kir4.1 modulators for pain or mental health disorders may have side effects. Kir4.1 is not only expressed in the nervous system like the brain, spinal cord, and ganglia, but also in other organs, such as the kidneys, inner ears, and eyes, involved in regulating K^+^ homeostasis, membrane potential, and healing processes [83,179,180]. The treatment of depression typically involves the inhibition of Kir4.1 function in the brain. However, insufficient drug selectivity may result in adverse effects resembling aspects of EAST/SeSAME syndrome, including renal tubulopathy (metabolic alkalosis, hypomagnesemia, hypokalemia, and hypocalciuria) [179], hearing loss [83], retinal edema, and elevated intraocular pressure [180]. Furthermore, Kir4.1 exhibits region-specific expression patterns within the brain, and its functional roles may vary across different brain areas [55,59], which necessitates even higher selectivity in drug delivery targeting Kir4.1.

Developing region-specific drug delivery systems holds promise in addressing this problem. Firstly, the application of an Implantable Intrathecal Drug Delivery System (IDDS) significantly alleviated cancer-related pain by directly delivering medication to the subarachnoid space. Due to the substantial reduction in dosage, the toxicity scores of patients in the IDDS group decreased by 50% compared to the control group, with noticeable reductions in fatigue and consciousness suppression [181]. Furthermore, this method has been reported to improve symptoms such as anxiety, depression, and sleep disturbances in cancer patients [182]. Secondly, nasal drug delivery offers several advantages that make it an alternative therapy for treating depression and anxiety. It allows for direct action on the brain and accommodates the transport of larger molecules (up to approximately 1000 Da). This route avoids hepatic metabolism, minimizes side effects associated with systemic circulation, reduces the required drug dosage, and is non-invasive [183]. In a randomized, double-blind, saline-controlled, crossover trial involving 20 patients with major depression, the Montgomery–Asberg Depression Rating Scale scores in the intranasal ketamine group were 7.6 points lower than those in the placebo group, and anxiety levels were significantly reduced [184]. Thirdly, targeted molecular recognition technologies utilize drug carriers such as nanoparticles [185], antibodies, or peptides [186] to recognize specific receptors and concentrate the drug at the target site. In particular, combining nanotechnology with nasal drug delivery further enhances the efficacy of medications. For instance, a study involving a fluoxetine nasal formulation based on lipid nanoparticles demonstrated longer immobility times in the FST compared to oral administration [187]. This may be attributed to the fact that intranasally administered drugs typically reach their maximum concentration in the brain within the first 15 min, whereas the test was conducted an hour post-treatment. However, the intranasal group exhibited a higher percentage of inhibition in the Marble-Burying Test (MBT), indicating a greater alleviation of anxiety-like behaviors [187]. In summary, the above approaches may help resolve the current challenges of specificity, pharmacokinetic issues, selectivity, side effects, and safety in designing specific Kir4.1 modulators.

### 5.3. Future Research Directions Based on Kir4.1

Gene therapy and precision medicine strategies have increasingly emerged as prominent research focal points. Gene therapy strategies targeting Kir4.1 frequently aim to regulate its expression to counteract disease mechanisms. For pain management, upregulation of Kir4.1 expression is generally necessary [60], whereas in the case of depression, a downregulation is more appropriate [130]. Upregulation is generally achieved by delivering exogenous genes through viral vectors, while downregulation is facilitated using RNA interference technologies. Viral vectors can be categorized into AAV and lentivirus types, with the former being predominantly used. For instance, overexpression of Kir4.1 in the TG via AAV-Kir4.1 transfection improves the head-withdrawal threshold in mice subjected to CCI-ION and IANX treatments [80,114]. A critical aspect of gene therapy success lies in using specific promoters to ensure restricted transgene expression; the gfaABC1D promoter specifically targets GFAP-expressing astrocytes [188]. Conversely, knockdown or knockout of Kir4.1 may also provide therapeutic benefits. Injection of shRNA into the HIP or LHb to silence Kir4.1 significantly alleviates depression-like phenotypes [52,59,131]. Moreover, silencing Kir4.1 expression with siRNA promotes BDNF secretion while also inhibiting glutamate uptake [30,46], both of which enhance neuronal excitability. However, these approaches have limitations. AAV vectors have restricted cargo capacities, and short, single-stranded RNA exhibits limited half-life, necessitating frequent administration [189,190].

Pain and mental health disorders, including depression, are characterized by complex neurobiological mechanisms in which Kir4.1 represents just one of many components. Pharmacogenetic-guided medication selection has been shown to significantly improve outcomes for patients with depression and anxiety [191]. In future clinical trials, patients may be stratified through genetic testing—such as screening for Kir4.1 mutations or polymorphisms. This approach will help identify cases where Kir4.1 dysfunction plays a central role in pathogenesis or where patients are more likely to respond favorably to therapies targeting this channel. Such approaches allow for the design of personalized treatment strategies tailored to the specific etiological factors in individual patients, enhancing therapeutic efficacy while minimizing the risk of adverse effects. Despite these promising approaches, the clinical application of therapies targeting Kir4.1 presents several challenges, including limited specificity, pharmacokinetic constraints, difficulties in crossing the blood–brain barrier, achieving targeted tissue delivery, and potential safety concerns. Addressing these challenges may require innovative solutions such as chemical modifications, structure-based drug design, and precision medicine strategies, which together could improve therapeutic outcomes and overcome current limitations. Furthermore, high-resolution structural analyses and computational molecular docking provide valuable insights for future structure-based rational drug design targeting Kir4.1. Combination therapies may also represent an effective strategy to maximize the therapeutic potential of Kir4.1 modulation, and well-designed randomized controlled trials will be required to provide further validation. For instance, it will be important to compare the efficacy of a monotherapy group versus a combination therapy group and systematically assess the full spectrum of adverse reactions.

## 6. Conclusions

Kir4.1, predominantly expressed in glial cells such as astrocytes and SGCs, plays a pivotal role in maintaining extracellular potassium and glutamate homeostasis within the nervous system. Dysregulation of Kir4.1 alters neuronal excitability and synaptic transmission, which are fundamental mechanisms underlying chronic pain and mental health disorders like depression and anxiety. In chronic pain, downregulation of Kir4.1 impairs potassium buffering and glutamate clearance, leading to heightened neuronal excitability in both peripheral and central sensitization. In mental health disorders, aberrant Kir4.1 function affects the secretion of neurotrophic factors, such as BDNF, and modulates neuroinflammatory pathways, contributing to mood dysregulation. Thus, Kir4.1 acts as a core regulatory element in the comorbidity mechanisms of chronic pain and mental health disorders by influencing glial function and neuronal activity.

Targeting Kir4.1 offers significant therapeutic potential for treating chronic pain and mental health disorders. Pharmacological modulation of Kir4.1, through selective inhibitors or activators, may restore ionic and neurotransmitter balance, alleviating symptoms associated with these conditions. Antidepressants like tricyclics and SSRIs have been found to inhibit Kir4.1, suggesting a novel mechanism underlying their therapeutic effects. Future research should focus on developing highly selective Kir4.1 modulators with optimal pharmacokinetic properties and the ability to cross the blood–brain barrier. Gene therapy approaches to precisely regulate Kir4.1 expression in specific glial populations also hold promise. Advancements in these areas could lead to novel, effective treatments that address both chronic pain and comorbid mental health disorders, ultimately improving patient outcomes and quality of life.

## Figures and Tables

**Table 1:** Types of the inwardly rectifying potassium channel.

Subfamily	Kir Channel Subtype
G protein-gated channels	Kir3.4, Kir3.2, Kir3.3, Kir3.1
ATP-sensitive channels	Kir6.1, Kir6.2
Classical channels	Kir2.2, Kir2.3, Kir2.1, Kir2.4
K^+^ transport channels	Kir1.1, Kir7.1, Kir5.1, Kir4.1, Kir4.2

## Data Availability

No datasets were generated or analyzed during the current study.

## Abbreviations

MDD	Major depressive disorder
ACC	Anterior cingulate cortex
mPFC	Medial prefrontal cortex
DRN	Dorsal raphe nucleus
NAc	Nucleus accumbens
CeA	Central amygdala
VS	Ventral striatum
SGCs	Satellite glial cells
CNS	Central nervous system
Kir	Inwardly rectifying potassium
KATP	Adenosine triphosphate-sensitive potassium
DRG	Dorsal root ganglion
FST	Forced swim test
TST	Tail suspension test
Kir4.1	Inwardly rectifying potassium channel 4.1
BDNF	Brain-derived neurotrophic factor
TM	Transmembrane
HIP	Hippocampus
OLs	Oligodendrocytes
OPCs	Oligodendrocyte precursor cells
[K^+^]_o_	Extracellular K^+^ concentration
EAATs	Excitatory amino acid transporters
AQP4	Aquaporin-4
Glu	Glutamate
Gln	Glutamine
AP	Action potential
VGPC	Voltage-gated potassium channel
KAR	Kainic acid receptor
NMADR	N-methyl-D-aspartate receptor
AMPAR	α-amino-3-hydroxy-5-methyl-4-isoxazolepropionic acid receptor
mGluR	Metabotropic glutamate receptor
GDNF	Glial cell line-derived neurotrophic factor
LPS	Lipopolysaccharide
RACK1	Receptor for activated C kinase 1
cKO	Conditional knockout
CPG	Central pattern generator
LHb	Lateral habenula
RTN	Retrotrapezoid nucleus
MBP	Myelin basic protein
tMCAO	Transient middle cerebral artery occlusion
TG	Trigeminal ganglion
LC	Locus coeruleus
BNST	Bed nucleus of the stria terminalis
WKY	Wistar-Kyoto
TMJ	Temporomandibular joint
TCAs	Tricyclic antidepressants
CCI-ION	Chronic constriction injury of the infraorbital nerve
CFA	Complete Freund’s adjuvant
LH	Lateral hypothalamus
IANX	Inferior alveolar nerve transection
GPR37L1	G protein-coupled receptor 37-like 1
MaR1	Pro-resolving lipid mediator maresin 1
MeCP2	Methyl-CpG-binding protein 2
PWSI	Post-weaning social isolation
dBNST	Dorsal bed nucleus of the stria terminalis
NSF	Novelty-suppressed feeding
CORT	Corticosterone-induced depression
SSRIs	Selective serotonin reuptake inhibitors
IC50	Half-maximal inhibitory concentration
MBT	Marble-burying test
IDDS	Implantable Intrathecal Drug Delivery System
AAV	Adeno-associated virus
